# Using Information Available at the Time of Donor Offer to Predict Kidney Transplant Survival Outcomes: A Systematic Review of Prediction Models

**DOI:** 10.3389/ti.2022.10397

**Published:** 2022-06-23

**Authors:** Stephanie Riley, Qing Zhang, Wai-Yee Tse, Andrew Connor, Yinghui Wei

**Affiliations:** ^1^ Centre for Mathematical Sciences, School of Engineering, Computing and Mathematics, University of Plymouth, Plymouth, United Kingdom; ^2^ Department of Renal Medicine, South West Transplant Centre, University Hospitals Plymouth NHS Trust, Plymouth, United Kingdom

**Keywords:** kidney transplantation, prognosis, systematic review, clinical prediction model, decision-making, kidney transplant outcomes, prediction tool

## Abstract

Statistical models that can predict graft and patient survival outcomes following kidney transplantation could be of great clinical utility. We sought to appraise existing clinical prediction models for kidney transplant survival outcomes that could guide kidney donor acceptance decision-making. We searched for clinical prediction models for survival outcomes in adult recipients with single kidney-only transplants. Models that require information anticipated to become available only after the time of transplantation were excluded as, by that time, the kidney donor acceptance decision would have already been made. The outcomes of interest were all-cause and death-censored graft failure, and death. We summarised the methodological characteristics of the prediction models, predictive performance and risk of bias. We retrieved 4,026 citations from which 23 articles describing 74 models met the inclusion criteria. Discrimination was moderate for all-cause graft failure (C-statistic: 0.570–0.652; Harrell’s C: 0.580–0.660; AUC: 0.530–0.742), death-censored graft failure (C-statistic: 0.540–0.660; Harrell’s C: 0.590–0.700; AUC: 0.450–0.810) and death (C-statistic: 0.637–0.770; Harrell’s C: 0.570–0.735). Calibration was seldom reported. Risk of bias was high in 49 of the 74 models, primarily due to methods for handling missing data. The currently available prediction models using pre-transplantation information show moderate discrimination and varied calibration. Further model development is needed to improve predictions for the purpose of clinical decision-making.

**Systematic Review Registration:** https://osf.io/c3ehp/l.

## Graphical Abstract

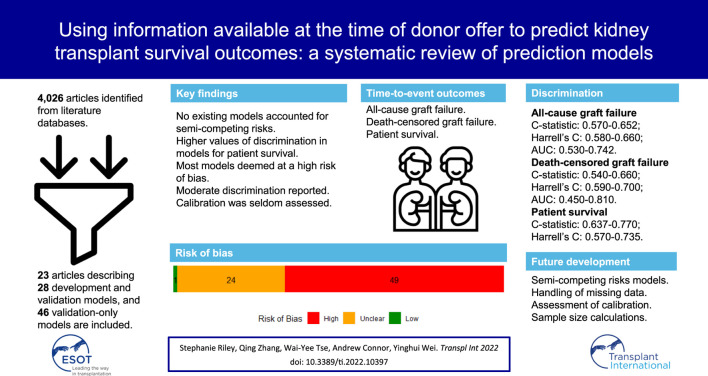

## Introduction

End-stage kidney disease (ESKD) is the most advanced stage of chronic kidney disease. Kidney transplantation is the optimal treatment for many patients with ESKD. In the UK, approximately 3,000 kidney transplants are performed every year, but the number of patients waiting for a transplant is around 5,000 (1). The success, in terms of efficacy and longevity, of an individual transplant will be influenced by a host of factors, some of which can be determined prior to transplantation. A balance must be struck to ensure maximal organ utilisation without compromising transplant outcomes. This is further complicated by the fact that “one size does not fit all”—the definition of a successful transplant will vary depending on the recipient and their clinical scenario. As such, every potential kidney transplant must be carefully considered in the context of the donor and recipient details.

In the UK donor organs are offered through a national donation system, which utilises an algorithm to balance patient priority and the intent to match immunological and additional parameters. The donor offers are reviewed by clinicians acting on behalf of the recipient and a prompt decision must be made to accept or reject each offer. Whether or not to accept a transplant offer remains a challenging clinical decision. Clinical prediction models that utilise information which would commonly be available to the clinician at the time of the donor kidney offer may help to inform the decision-making process.

The anticipated longevity of a kidney transplant is, of course, an important consideration for a clinician faced with the kidney donor acceptance decision. However, given that donor kidneys are a scarce resource and potential recipients must therefore sit on waiting lists, it is often appropriate to balance the anticipated longevity against the alternative of remaining on dialysis. As such, models that can predict graft survival outcomes would be of great clinical utility.

Prediction models have previously been developed for kidney transplant survival outcomes with the aim of advising clinicians at the time of the offer of a donor kidney. The number of articles related to clinical prediction models for kidney transplant survival outcomes is increasing year on year, suggesting a recognition of the clinical interest. The Kidney Donor Risk Index (KDRI) (2), Estimated Post Transplant Score (EPTS), Maryland Aggregate Pathology Index (MAPI) (3) and Living Kidney Donor Profile Index (LKDPI) (4) are commonly reported risk indices. The KDRI and EPTS are part of the kidney allocation system in the US.

The aforementioned risk indices were developed in the US population. A similar index has been produced in the UK (UK KDRI) (5), though is not widely used in practice. In the UK kidney allocation system NHS Blood and Transplant (NHSBT) use their own risk indices for donors and recipients (6). This is to help ensure that the pool of donor kidneys is utilised to best effect. Through this system, for example, younger recipients will typically receive offers of kidneys from younger donors (in order to optimise the chances of transplant longevity) whilst a greater tolerance of less favourable immunological matches will be accepted for older recipients (in order to maximise offers for a cohort in whom immunological matching is of slightly less importance).

We identified two systematic reviews exploring existing prediction models for kidney transplantation. Kaboré et al. (7) reviewed prediction models for graft outcomes published between 2005 and 2015, while Senanayake et al. (8) reviewed machine learning methods to predict graft failure, delayed graft function (DGF) and acute graft rejection. Since only machine learning models were eligible, their review excluded articles that used the Cox model, which is the model most used for time-to-event analyses.

Both reviews allowed the inclusion of predictors that only become available after transplantation, such as whether patients experienced DGF. To our knowledge this is the first review to focus only on models that could aid clinical decision-making at the time of the donor offer.

In this systematic review we aim to identify, appraise and summarise existing clinical prediction models for kidney transplant survival outcomes. Only prediction models that use information available at the time of the single kidney-only offer were included, allowing us to focus on models with the most clinical utility.

## Methods

We prospectively developed a protocol which is publicly available from OSF (9). The findings of this review are reported in accordance with the Preferred Reporting Items for Systematic reviews and Meta-Analyses (PRISMA) statement (10).

### Eligibility Criteria

We included studies with adult recipients (aged 18 years or older) of single, kidney-only transplants. No restrictions were placed on donor type.

No limit was set on publication date. Only full texts published in English were eligible. Conference abstracts without full text were excluded from review.

The outcomes of interest were one or both of the following outcomes, time to graft failure and time to death at any time point following kidney transplantation. Models that did not account for time-to-event information were excluded.

We considered prediction models that make use of information available at the time of a donor kidney offer to inform the acceptance decision. Prediction models developed using predictors that only become available after transplantation were not included, as the decision would have been made by that time.

We included studies which were developed and validated for the outcomes of interest, and validation-only studies which validated existing models developed from independent cohorts. Any measure of predictive performance, such as calibration or discrimination, that was reported alongside a model was considered a form of validation. Validation-only model refers to the case where the current study validates an existing model.

### Information Sources and Search Strategy

Electronic databases Embase, MEDLINE and Web of Science were searched from their respective dates of inception up to April 8th^,^ 2021. The search strategy is presented in Supplementary Table S1.

All citations from the search results were exported to Endnote, where duplicates were automatically removed from review. Titles and abstracts of all records were independently screened against the above eligibility criteria by two reviewers (SR and QZ) and managed through Rayyan (11). A third reviewer (YW) also independently screened 10% of the titles and abstracts. Two reviewers then independently reviewed full-text reports to assess eligibility (SR and QZ). Any discrepancies were resolved through discussion.

### Data Extraction

Data were extracted from eligible articles according to the Critical Appraisal and Data Extraction for Systematic Reviews of Prediction Modelling Studies (CHARMS) checklist (12). The full list of data extracted are given in Supplementary Table S2. Data were extracted independently by two reviewers (SR and YW) and any discrepancies were resolved through discussion.

### Risk of Bias

We assessed the risk of bias (RoB) in individual models using the Prediction Model Risk of Bias Assessment Tool (PROBAST) (13). Two reviewers (SR and YW) independently determined the RoB of each model and any disagreements were resolved by discussion.

### Outcomes

#### All-Cause Graft Failure

All-cause graft failure, as a composite outcome, is defined as the earliest time to graft failure or death.

#### Death-Censored Graft Failure

Death-censored graft failure considers the time until graft failure, but patients are censored at the time of death. Graft failure and death are semi-competing events (14). Semi-competing events arise when a terminal event precludes a non-terminal event, but not vice-versa (15).

#### Death

This measures time to recipient death, of any cause, as the outcome of interest.

### Analysis

#### Study Characteristics

We summarised the year of publication, geographical location, model type, and model being validated. We explored the discrimination measures by sample size and predictor type (donor, recipient, transplant, or combination of these). For each outcome, we summarised the type of predictors, modelling methods, and methods for handling missing data.

#### Measures of Model Performance

Model performance was evaluated by calibration and discrimination. Calibration assesses the agreement between observed and predicted risk and is often reported through a calibration plot. Discrimination measures a model’s ability to separate recipients who will experience the outcome event versus those who will not. It is often measured using Harrell’s C statistic, area under receiver operating characteristic curve (AUC) or time-dependent AUC, which account for the censoring of the time-to-event outcome. When a model is developed and internally validated in the same dataset it understandably performs well. Methods to correct for this optimism can be administered using bootstrapping, and resulting measures are referred to as optimism-corrected (16). Where studies did not explicitly state that the C-statistic was adapted for censoring, we elected to report the terminology used in the original articles.

## Results

**FIGURE 1:**
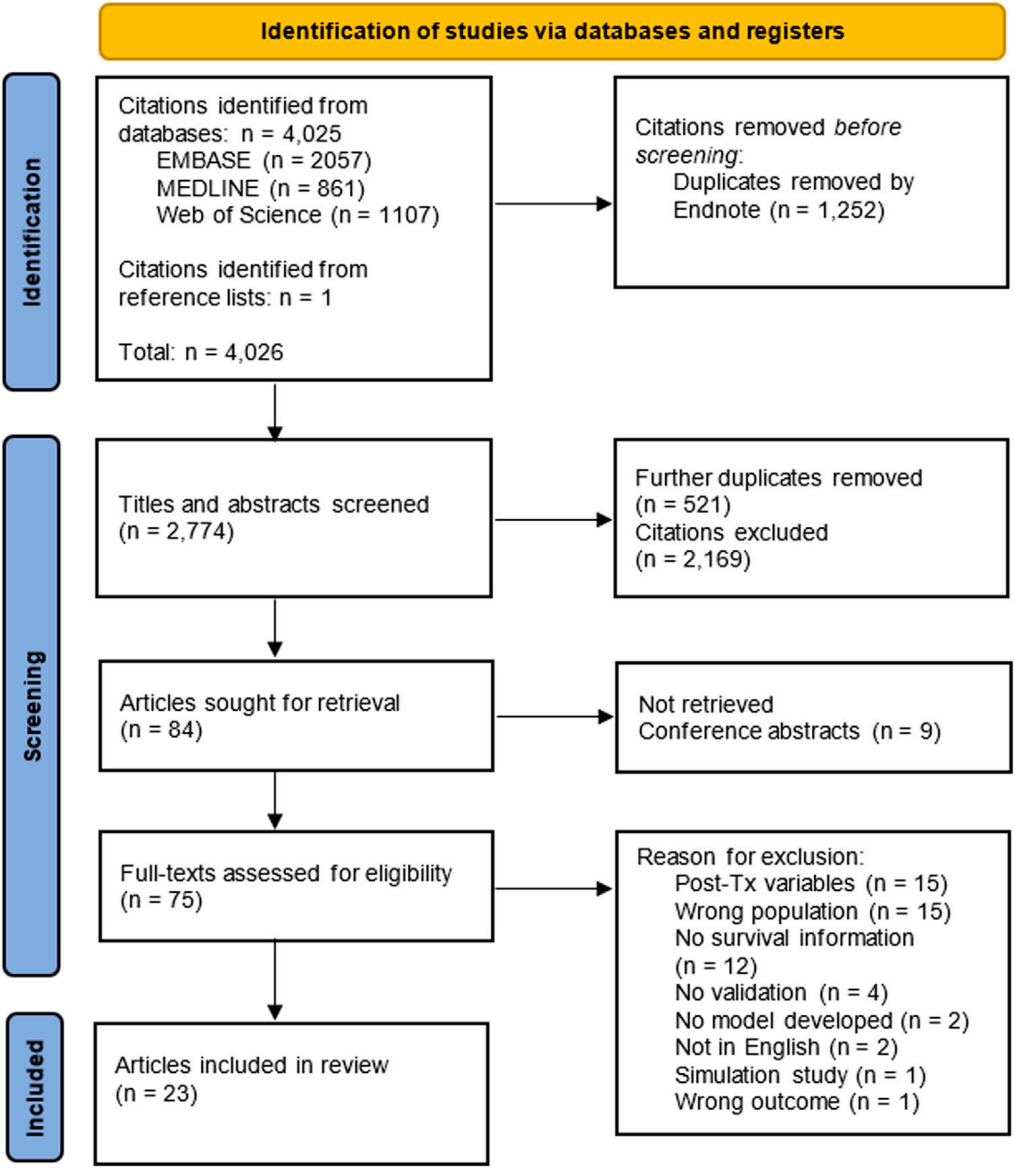
Flowchart of articles eligible for inclusion in the systematic review. Each database was searched from their respective date of inception until April 8th 2021.

We retrieved 4,025 citations from three databases through our search and identified one record related to one of the conference abstracts we screened. After the initial screening of titles and abstracts, 75 articles were retrieved for full-text review. Of these records, 23 articles describing 74 models met the inclusion criteria (3–5, 17-36) (Figure 1).

### Characteristics of Included Studies

Of the 74 eligible models, 28 developed and validated a clinical prediction model for our outcomes of interest. The remaining 46 models validated the performance of an existing model in an independent cohort. Articles were published between 2005 and 2020; fifteen of the twenty-three articles (65.22%) were published after 2015. Twelve articles used data of recipients from the United States, four from mainland Europe, three from Canada, two from Australia and New Zealand, and one each from the United Kingdom and Thailand. Characteristics of included studies for each model are available in Supplementary Tables S3–S6.

In the 28 development and validation models, 27 used the Cox proportional hazards model, while one (17) used a survival random forest. Only eight of the Cox models assessed the proportional hazards assumption.

**FIGURE 2:**
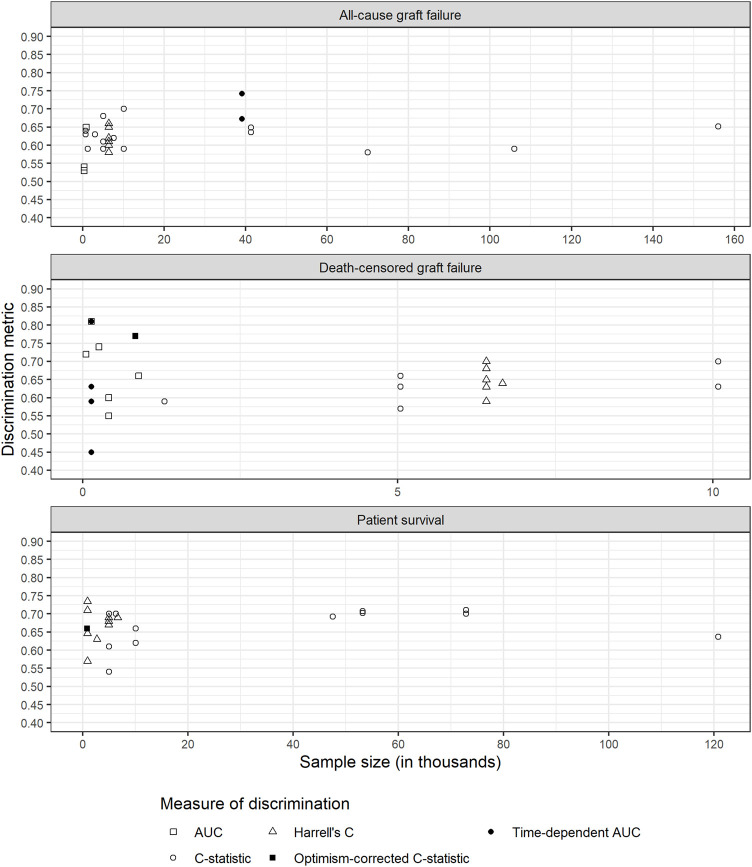
Discrimination metrics against sample size for each outcome. AUC: area under receiver operating characteristic curve; C-statistic: concordance statistic; Harrell’s C: adapts the C-statistic to account for censoring; Optimism-corrected C-statistic: measures the C-statistic while accounting for optimism in model performance; Time-dependent AUC: a measure of the AUC at specified timepoints since time origin.

There was considerable variability in sample sizes used for models (Table 1; Figure 2). In general, models performing validation alone tended to have smaller sample size. Models with smaller sample sizes did not have noticeably poorer discrimination for any of the outcomes (Figure 2).

**TABLE 1:** Summary of sample size used in models for each outcome and model type.

	Number of models	Range	Median	Mean	SD
All-cause graft failure					
Development and validation	11	785–156,069	39,108	41,127	48,719
Validation only	15	416–69,994	5,042	8,641	17,141
Death-censored graft failure					
Development and validation	5	259–10,086	6,662	5,586	4,811
Validation only	19	56–6,405	1,299	3,017	2,909
Patient survival					
Development and validation	11	837–120,818	47,535	41,319	38,270
Validation only	11	935–5,042	4,983	3,323	2,007

Two models with other outcomes which do not fall into the above definitions have sample size of 20,085 and 2,734, respectively.

We considered three types of predictors, donor characteristics, recipient characteristics and transplant process. We found no clear evidence that the type of predictors was associated with better discrimination for any outcome (Supplementary Figures S1–S3). Clayton et al. (21) validated the US and UK KDRI, while also adjusting for recipient characteristics and transplant process. Those with higher values of discrimination (models 9 and 12) were adjusted for other donor, recipient and transplant related predictors. This was also observed by Molnar et al. (28). However, this increase could simply be due to having more variables in the model.

Nine of the 28 development and validation models (4, 24, 27, 28) were available in the form of an online tool or calculator. One of the models (32) was presented in the form of a nomogram and another (17) as a contour plot of survival probability.

Commonly validated risk indices, as described in Table 2, included the KDRI, EPTS, UK KDRI, LKDPI, and MAPI. Other models validated included those developed by Kasiske et al. (27), Nyberg et al. (37) and Remuzzi et al. (38).

**TABLE 2:** Summary of commonly reported risk indices for predicting kidney transplant survival outcomes.

Model	Donor	Recipient	Transplant organ/process	Histopathology	Validation studies
EPTS	NA	Age;	NA	NA	(22)
		Diabetes status;			(23)
		Prior solid organ transplants; Time on dialysis			(28)
LKDPI	Age; eGFR;	Sex (compared to donor); Weight (relative to donor weight)	Number of HLA mismatch at HLA-B and HLA-DR; ABO compatibility	NA	(30)
	BMI;				
	Ethnicity;				
	History of cigarette use;				
	Systolic blood pressure;				
	Sex;				
	Weight				
MAPI	NA	NA	NA	Arteriolar hyalinosis;	(25) (29)
				Glomerulosclerosis;	
				Periglomerular fibrosis;	
				Scar	
				Wall-to-lumen ratio interlobular arteries	
UK KDRI	Age;	NA	NA	NA	(21)
	Days in hospital;				
	History of hypertension;				
	Use of adrenaline;				
	Weight				
US KDRI	Age;	NA	Cold ischaemic time; Double kidney transplant; En-bloc transplant; Number of HLA mismatch at HLA-B and HLA-DR	NA	(20)
	Cause of death;				(21)
	DCD;				(23)
	Diabetes status;				(25)
	Ethnicity;				(4)
	HCV status;				(30)
	Height				(5)
	History of hypertension				(35)
	Serum creatinine				
	Weight				

BMI, body mass index; DCD, deceased cardiac donor; eGFR, estimated glomerular filtration rate; HCV, hepatitis C virus; HLA, human leukocyte antigen.

### Risk of Bias

**FIGURE 3:**
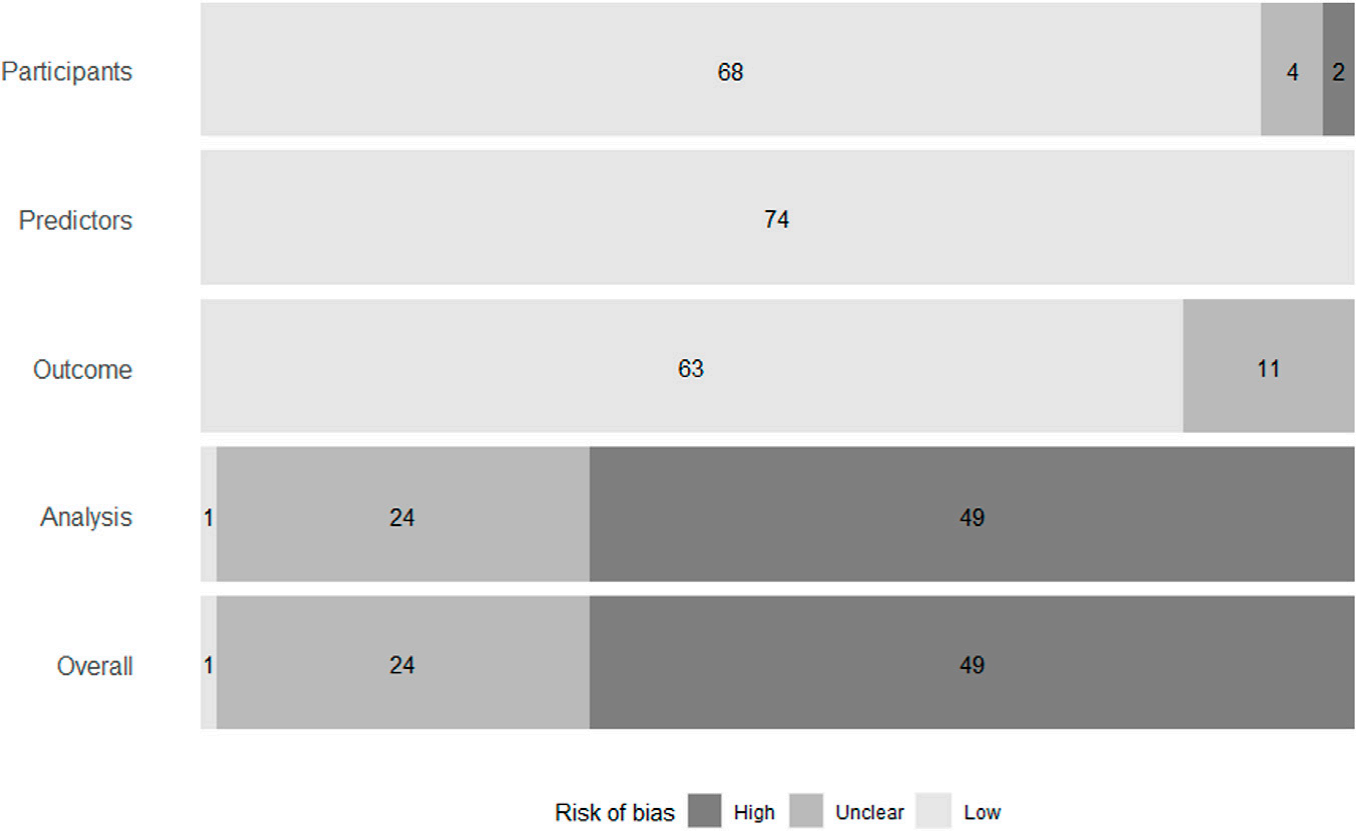
Summary of risk of bias of models in individual domains, and overall. Risk of bias was assessed using the Prediction Model Risk of Bias Assessment Tool (13).

The overall RoB was high in 49 of the 74 models, unclear in 24, and low in only one (Figure 3). Of those that were considered a high RoB overall, all of them were at a high RoB in the analysis domain.

Sample size was reported for all models. However, the number of events were not reported for 37 of the models, therefore it was unclear whether there were a reasonable number of participants with the outcome.

Missing data were not discussed for 12 models. For models that did discuss missing data, 20 performed their analysis based only on those patients that did not have any missing data. This is called a complete-case analysis. All included models reported some measure of discrimination, but calibration was only reported for 13 models. Some models reported the C-statistic but did not discuss whether they had adapted it to account for censoring. This also contributed to a lack of clarity on the suitability of performance measures.

Twenty-two of the 28 development and validation models avoided univariable selection, reducing the possibility of bias in the analysis domain. Sixteen models did not account for overfitting or optimism, rendering them a high RoB.

### Development and Validation Models

#### All-Cause Graft Failure

All-cause graft failure was reported in 11 of the 28 development and validation models. Summary data for each model with this outcome are shown in Supplementary Table S3.

In eight models, only deceased donor information was used. Three models utilised a combination of living and deceased donors.

Four models performed a complete-case analysis and two models used multiple imputation (39) to handle missing data. Three models imputed values based on mean or median, and two models assigned missing values to a missing category.

**FIGURE 4:**
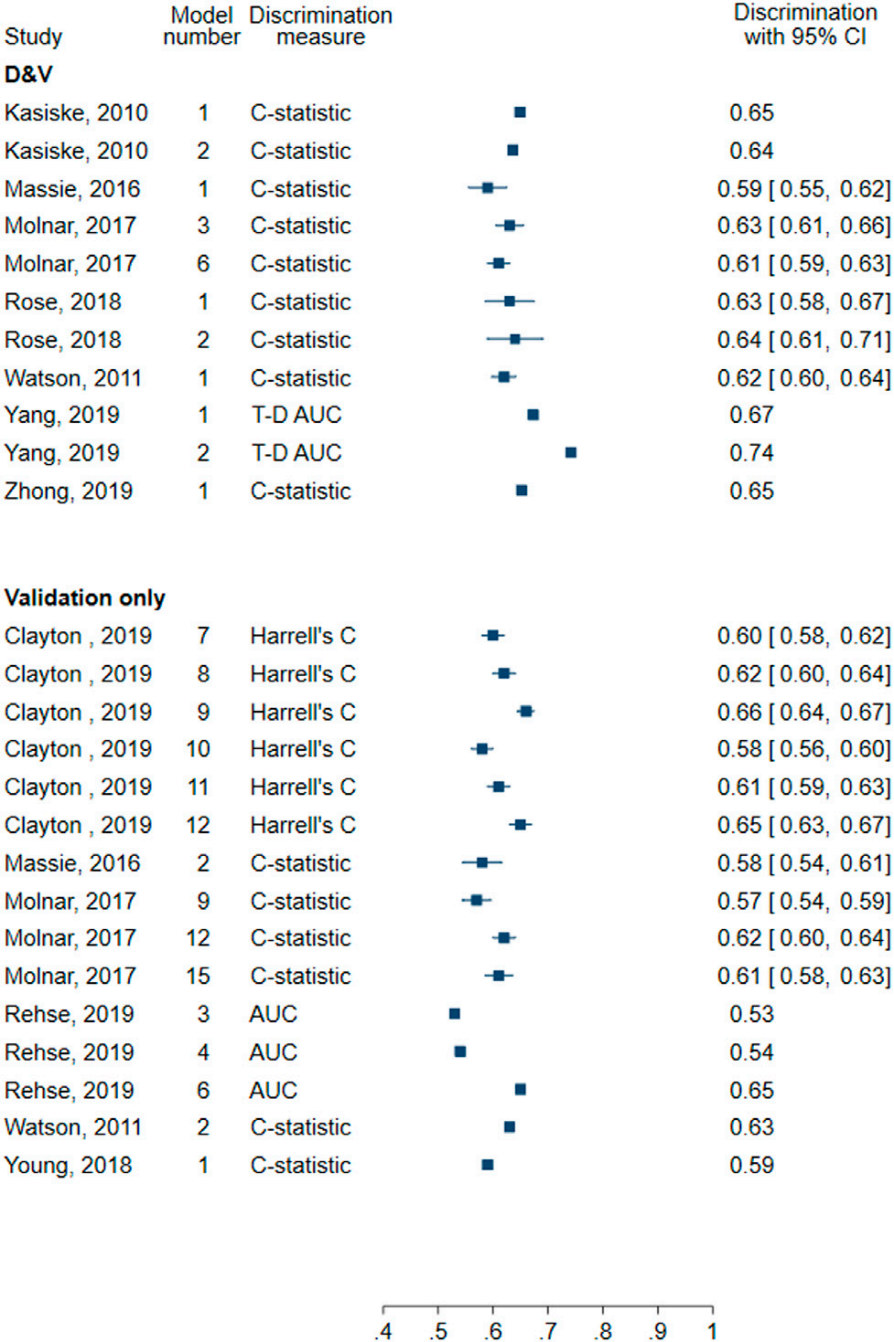
Forest plot of discrimination in models for all-cause graft failure. D&V: Development and validation; AUC: area under receiver operating characteristic curve; C-statistic: concordance statistic; Harrell’s C: adapts the C-statistic to account for censoring; T-D AUC: Time-dependent AUC, a measure of the AUC at specified timepoints since time origin.

All models assessed discrimination. Discrimination measures reported included nine C-statistics (0.59–0.652) and two time-dependent AUC at 20 years (0.673 and 0.752) (Figure 4). In four models that also assessed calibration, two did so using a calibration plot and the remaining two reported the calibration slope (1.04 each).

#### Death-Censored Graft Failure

In one of the five models for death-censored graft failure the eligible population was deceased donor kidney recipients whilst in one model it was living donor recipients (Supplementary Table S4). Three models utilised a combination of both living and deceased donors.

For death-censored graft failure, four models used multiple imputation and one failed to report any methods for handling of missing data.

**FIGURE 5:**
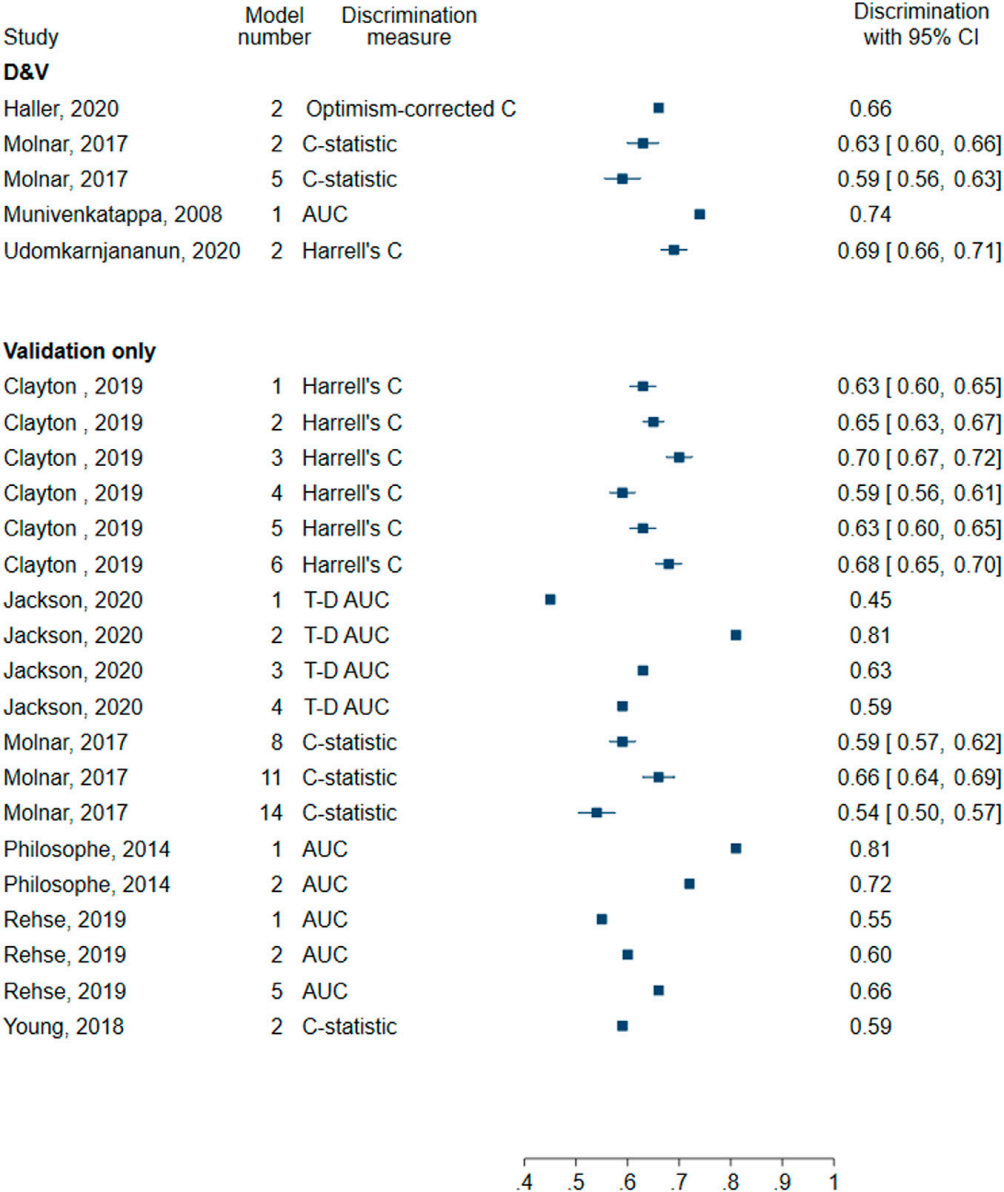
Forest plot of discrimination in models for death-censored graft failure. D&V: Development and validation; AUC: area under receiver operating characteristic curve; C-statistic: concordance statistic; Harrell’s C: adapts the C-statistic to account for censoring; Optimism-corrected C-statistic: measures the C-statistic while accounting for optimism in model performance; T-D AUC: Time-dependent AUC, a measure of the AUC at specified timepoints since time origin.

All models included at least one measure of discrimination and four evaluated the calibration. Discrimination measures reported included Harrell’s C (0.69), AUC (0.74), C-statistic (0.59, 0.63), and optimism-corrected C-statistic (0.66) (Figure 5). Three models graphically assessed calibration and one used the Hosmer-Lemeshow test.

#### Patient Survival

Only one of the 11 models utilised living donors, whilst six used deceased donor transplant data and four considered a combination of living and deceased donors (Supplementary Table S5).

Eight models handled missing data using multiple imputation and one used single imputation. One model undertook a complete-case analysis and handling of missing data was not reported for one model.

**FIGURE 6:**
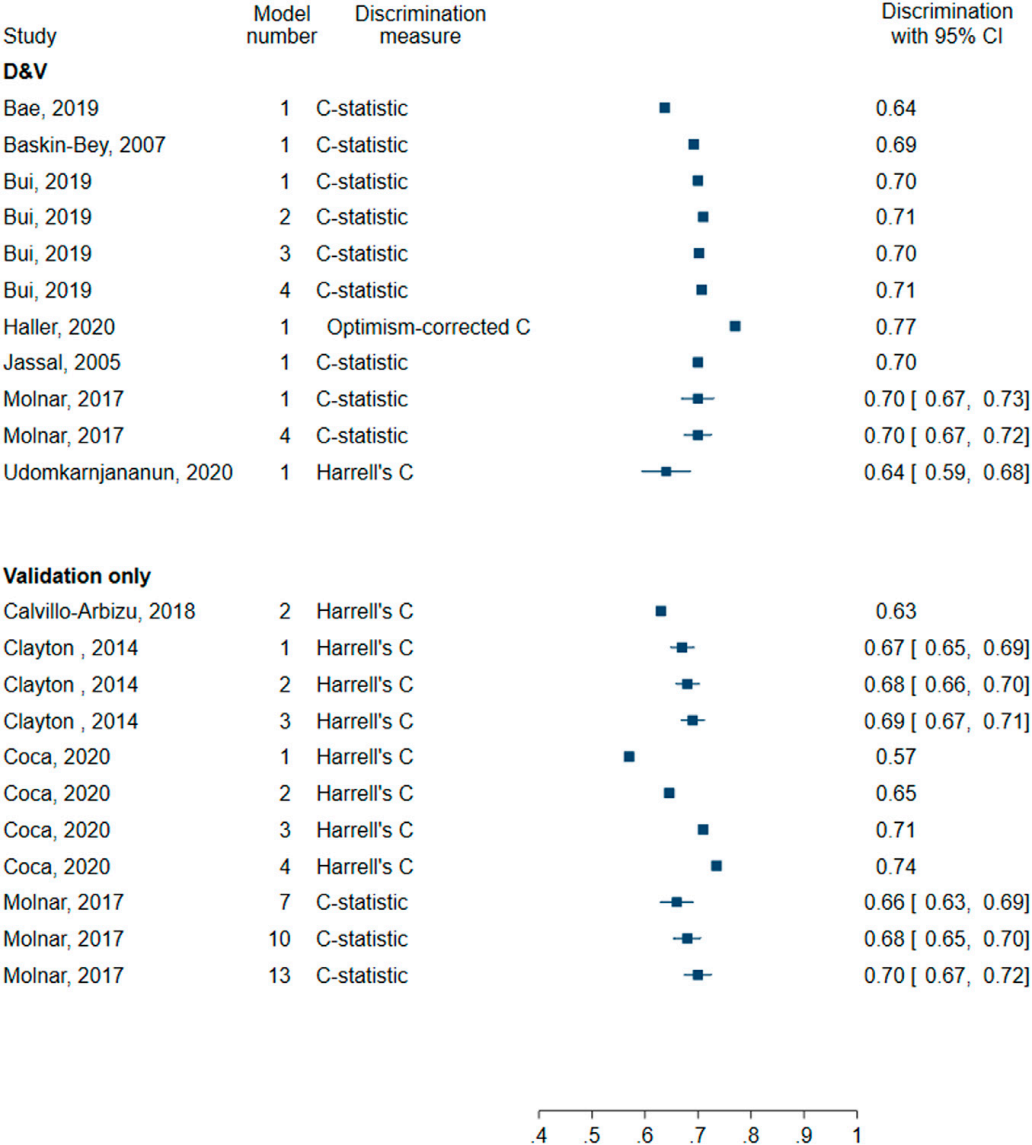
Forest plot of discrimination in models for patient survival. D&V: Development and validation; C-statistic: concordance statistic; Harrell’s C: adapts the C-statistic to account for censoring; Optimism-corrected C-statistic: measures the C-statistic while accounting for optimism in model performance.

The C-statistic was the most used measure of discrimination (9 models) with reported values between 0.637 and 0.71 (Figure 6). Other measures included Harrell’s C (0.64) and optimism-corrected C-statistic (0.77). Calibration was also assessed in four models, three of which presented a calibration plot while the other performed the Hosmer-Lemeshow test.

### Validation-Only Models

#### All-Cause Graft Failure

All-cause graft failure was reported in 15 of the 46 validation-only models (Supplementary Table S3).

In 10 models, only deceased donor information was used and only living donor data in two models. The remaining three models utilised a combination of living and deceased donors.

Seven conducted a complete-case analysis. Three models used multiple imputation to handle missing data. Two models imputed values based on mean or median. For two models it was unclear how missing data were handled and one model did not discuss missing data.

All models assessed discrimination. Seven assessed discrimination using Harrell’s C (0.55–0.66) and six reported the C-statistic (0.57–0.63) (Figure 4). Two models used the AUC (0.53–0.65). No models assessed calibration.

#### Death-Censored Graft Failure

In 14 of the 19 validation-only models the eligible population was deceased donor kidney recipients whilst in two models it was living donor recipients (Supplementary Table S4). Three models utilised a combination of both living and deceased donors.

For death-censored graft failure, six models did a complete-case analysis, three models used multiple imputation and one used median imputation. For three models the methods for handling missing data were unclear, and six did not discuss missing data.

All models evaluated the discrimination, but none assessed the calibration. Four models reported the C-statistic (0.54–0.66) and six reported Harrell’s C (0.59–0.70). Five models assessed AUC (0.55–0.81), and four assessed time-dependent AUC evaluated 2 years following transplantation (0.45–0.81) (Figure 5).

#### Patient Survival

Eight out of 11 models used data from deceased donor transplant recipients and three used a combination of living and deceased donors (Supplementary Table S5).

Seven models handled missing data using multiple imputation and one conducted a complete-case analysis. Three models failed to discuss missing data.

Eight models assessed discrimination using Harrell’s C (0.57–0.735) and three using the C-statistic (0.66–0.70) (Figure 6). Calibration was not assessed in any of the validation-only models for patient survival.

## Discussion

### Principal Findings

Our review focussed on prediction models to inform the kidney donor acceptance decision. Thus, we only included models which used pre-transplantation information. The MAPI (3), for example, utilises histopathological data from pre-transplantation donor kidney biopsies to predict graft survival. However, clinicians in the UK would not typically have access to biopsy results at the time of offer this model has limited utility. The PreImplantation Trial of Histopathology In renal Allografts (PITHIA) (39) is ongoing and assesses whether pre-implantation biopsy analyses improve graft function. As such, there may be scope for the MAPI to be clinically useful.

Discrimination was well reported overall unlike calibration. Existing reviews also observed that calibration is poorly reported (40-43). Without both measures of performance, it is difficult to determine the predictive capability.

Twenty of the 28 development and validation models were developed in the US population, though the discrimination of these models remained similar in external validation in other countries. Overall performance of both development and validation-only models was most determined by measures of discrimination, such as the C-statistic, Harrell’s C, or AUC, which ranged between 0.59 and 0.77 for development and validation models, and 0.45 and 0.81 for validation-only models.

All included models considered the censoring of the time-to-event data using either Cox models or survival random forest. However, models for death-censored graft failure should have ideally considered the semi-competing events graft failure and death. Calvillo-Arbizu et al. (20) noted that death with a functioning graft is a competing event for graft failure but used this as part of the exclusion criteria. Methods such as Fine and Gray (44) and multistate models (45) can be used to account for semi-competing events without discarding the data.

The model by Haller et al. (24), reported optimism-corrected C-statistic 0.77 and showed good calibration. It predicted the survival of recipients of a living donor kidney using a combination of donor, recipient and transplant factors as predictors. However, it has not been externally validated, so its generalisability to other populations is not known.

The LKDPI by Massie et al. (4) predicted all-cause graft failure with C-statistic 0.59 (95% CI: 0.55–0.62). In external validation studies (30) conducted in Germany, the model continued to show moderate to poor discrimination. The development and validation model by Molnar et al. (28) predicting death-censored graft failure had similarly poor discrimination reporting a C-statistic 0.59 (95% CI: 0.56–0.63) but showed good calibration. No other article externally validated this model.

For deceased donors the model by Yang et al (34) reported time-dependent AUC equal to 0.742 for graft survival, but has not yet been validated externally.

The UK KDRI (5) for predicting transplant survival had moderate discrimination with a C-statistic of 0.62. External validation in Australia and New Zealand (21) reported Harrell’s C equal to 0.59 (95% CI: 0.56–0.61) and 0.58 (95% CI: 0.56–0.60) for predicting death-censored graft failure and all-cause graft failure, respectively.

Overall RoB was high in 49 out of 74 of the included models, largely due to the analysis methods. One such aspect was the handling of missing data. Twelve models did not discuss missing data at all, and twenty models handled missing data using a complete-case analysis. This analysis approach can lead to biased results (39) due to reduced sample size and increased risk of overfitting. Other methods, such as multiple imputation, are preferred over a complete-case analysis (46).

Overall, there was no clear indication whether the type of predictors affected discrimination (Supplementary Figures S1–S3). However, in individual articles we saw that models developed using combinations of type of predictors, rather than donor-only, showed better discrimination. Models with a small sample size relative to the number of predictors are more susceptible to overfitting (47), which can result in poorer predictive performance.

Sufficient sample size was rarely considered and was one contributing factor to models being deemed at a high RoB. Methods for calculating the effective sample size for the development of a prediction model for time-to-event outcomes have been proposed by Riley et al. (48). A sample size calculation is standard practice in clinical trials, and we believe this practice should cross over into prediction modelling.

### Strengths

To our knowledge this is the first review focusing on prediction models that only use information known prior to transplantation as predictors, and does not restrict to either regression or machine learning methods. Furthermore, we reviewed all articles published from the date of inception of each database, allowing us to maximise the number of articles included.

### Limitations

Our review was restricted to articles published in English. We focussed on models that would be of practical use at the time of an offer of a donor kidney. As such notable models including those by Loupy et al. (49) and Foucher et al. (50), which include post-transplantation information, were not eligible for our review. Based on the existing prediction models, we cannot conclude which methods work better than the other. This opens the opportunity for evaluation, application and testing a range of appropriate methods in future research.

## Conclusion

Development of clinical prediction models to inform organ acceptance decision-making should be driven by the clinical utility of such models. The currently available prediction models using pre-transplantation information provide moderate discrimination and varied calibration for patient and graft survival. Sample size calculations, handling of missing data and assessment of calibration are required, alongside better reporting of methods, to increase the quality of the studies. Opportunities to improve predictive performance include the identification of further important predictors and advancement of the development models by acknowledging the complex data such as semi-competing risks between graft failure and death. Until the predictive tools have the desirable performance, they have limited utility in clinical decision-making.

## Data Availability

Data extracted, risk of bias assessment and code are available from Github: https://github.com/Yinghui-Wei-team/methods-review-kidney-transplant-prediction.

## Abbreviations

AUC, area under receiver operating characteristic curve; CHARMS, Critical Appraisal and Data Extraction for Systematic Reviews of Prediction Modelling Studies; DGF, delayed graft function; EPTS, Estimated Post Transplant Survival; ESKD, end-stage kidney disease; KDRI, Kidney Donor Risk Index; LKDPI, Living Kidney Donor Profile Index; MAPI, Maryland Aggregate Pathology Index; NHSBT, National Health Service Blood and Transplant; PITHIA, PreImplantation Trial of Histopathology In renal Allografts; PRISMA, Preferred Reporting Items for Systematic reviews and Meta-Analyses; PROBAST, Prediction Model Risk of Bias Assessment Tool; RoB, risk of bias; UK KDRI, United Kingdom Kidney Donor Risk Index.
