# Supplementary material for: Using Information Available at the Time of Donor Offer to Predict Kidney Transplant Survival Outcomes: A Systematic Review of Prediction Models
Source: Transpl Int. 2022 Jun 23;35:10397. doi: 10.3389/ti.2022.10397 (PMC9259750; doi:10.3389/ti.2022.10397)
Supplement: Supplementary file 1 [file DataSheet1.pdf]

Supplementary material

Using information available at the time of donor offer to predict kidney transplant survival outcomes: a systematic review of prediction models

Search strategy

Key	Search term
**Embase**	
1	*kidney transplantation/ OR ((kidney OR renal) AND transplant*) NOT ((liver OR heart OR pancreas) ADJ1 transplant*).tw.
2	*graft failure/ OR ((graft OR allograft) ADJ1 (failure OR loss)).tw.<br>OR<br>(survival OR death OR mortality).tw.
3	(predict* OR prognos* OR risk).tw.<br>ADJ3<br>(tool* OR calculat* OR model* OR algorithm OR scor* OR index OR probabilit*).tw.
4	1 AND 2 AND 3
**MEDLINE**	
1	*Kidney Transplantation/ OR ((kidney OR renal) AND transplant*) NOT ((liver OR heart OR pancreas) ADJ1 transplant*).tw.
2	*Graft Survival/ OR ((graft OR allograft) ADJ1 (failure OR loss)).tw.<br>OR<br>(survival OR death OR mortality).tw.
3	(predict* OR prognos* OR risk).tw.<br>ADJ3<br>(tool* OR calculat* OR model* OR algorithm OR scor* OR index OR probabilit*).tw.
4	1 AND 2 AND 3
**Web of Science**	
1	ts= ((kidney OR renal) AND transplant*) NOT ((liver OR heart OR pancreas) NEAR/0 transplant*)
2	ts= ((graft OR allograft) NEAR/0 (failure OR loss))<br>OR<br>ts=(survival OR death OR mortality)
3	ts=(predict* OR prognos* OR risk)<br>NEAR/2<br>ts=(tool* OR calculat* OR model* OR algorithm OR scor* OR index OR probabilit*)
4	1 AND 2 AND 3

tw: terms are searched in titles and abstracts; ADJx: terms are adjacent with x-1 words between them; ts: terms are searched in titles and abstracts; NEAR/x: terms are adjacent with x words between them.

Table S1: Search strategy in each database from their respective dates of inception until April 8th 2021.

Data extraction

1. Source of data
2. Participants
    - 2.1 Description (e.g. location, participant age, participant sex)
    - 2.2 Study dates
3. Outcomes of interest
    - 3.1 Type and definition of outcome (death, graft failure)
    - 3.2 Prediction horizon
4. Candidate predictors
    - 4.1 Number, type (e.g. donor, recipient, transplant) and list of variables (e.g. donor/recipient age, HLA mismatch, cold ischaemia time)
    - 4.2 Incorporation of variables in modelling (e.g. dichotomised, continuous, linear/non-linear transformation)
5. Sample size
    - 5.1 Number of participants
    - 5.2 Number of outcome events
    - 5.3 Events per variable
    - 5.4 Sample size calculation performed
6. Missing data
    - 6.1 Number of participants with missing predictor or outcome values
    - 6.2 Number of missing data by variable
    - 6.3 Handling of missing values (e.g. complete-case analysis, imputation)
7. Model development
    - 7.1 Modelling methods (e.g. Cox model, flexible parametric survival model, competing risks model)
    - 7.2 Modelling assumptions satisfied
    - 7.3 Methods for selection of predictors variables for inclusion and during multivariate modelling
    - 7.4 Criteria used for selection of predictors
    - 7.5 Number of predictors in final model
    - 7.6 Shrinkage/penalty methods used (e.g. uniform, penalised, global)
8. Model performance
    - 8.1 Calibration (e.g. calibration plots/slope)
    - 8.2 Discrimination (e.g. C-statistic, Royston's D)
    - 8.3 Classification measures
9. Model evaluation
    - 9.1 Internal validation methods (e.g. development vs test set, bootstrap, cross validation)
    - 9.2 External validation methods (e.g. different geographical location or time period)
10. Results
    - 10.1 Final model presented including coefficients estimated, baseline survival etc.
    - 10.2 Any alternative presentation of model (e.g. web-based prediction tool, conversion to risk score, nomogram)
11. Interpretation and discussion
    - 11.1 Intended use for model (e.g. clinical utilisation)
    - 11.2 Comparison with other studies

Table S2: Items extracted from eligible studies. Table built based on the CHARMS checklist.

Summary of discrimination by predictor: all-cause graft failure

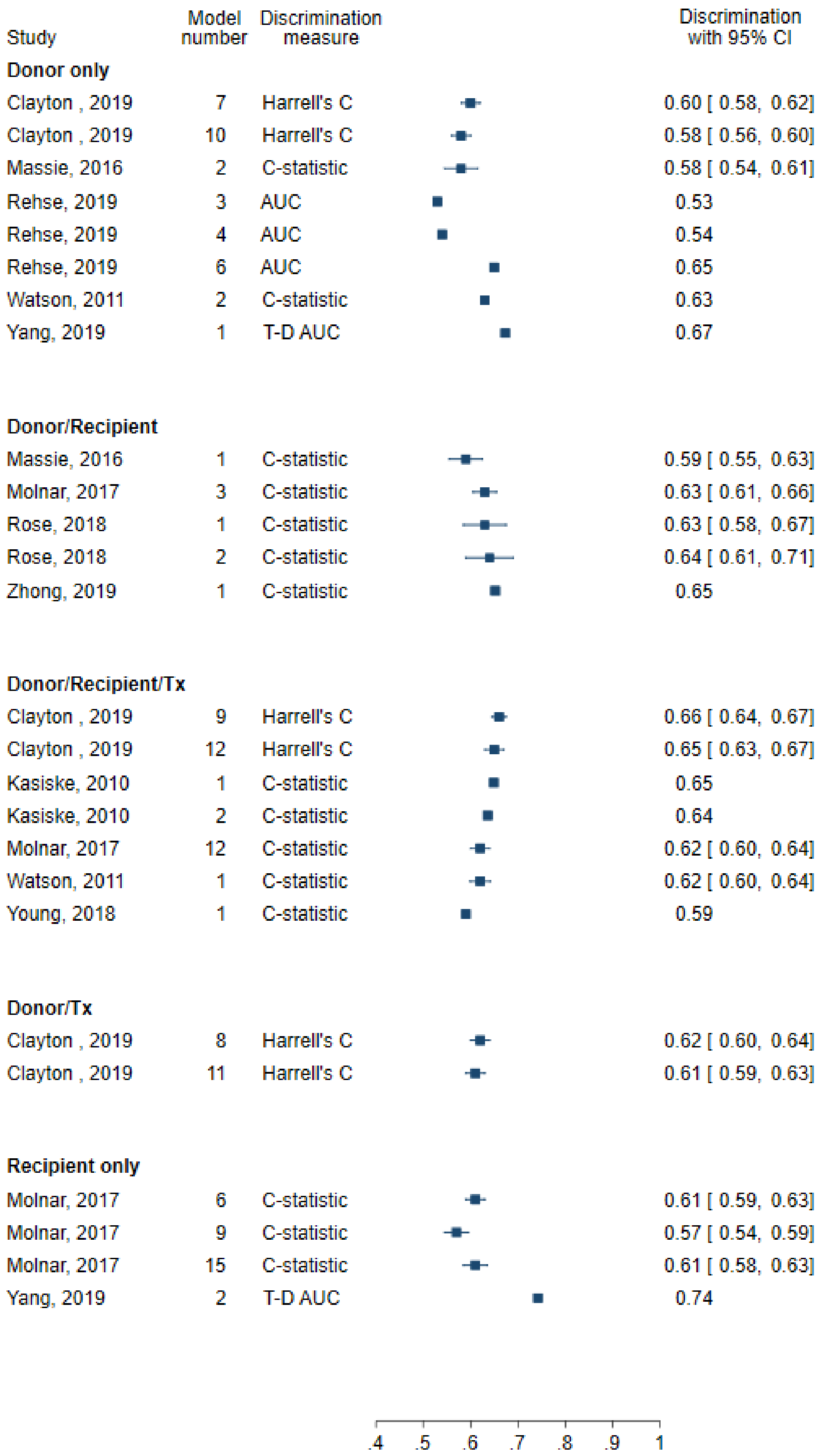

Figure S1: Discrimination of models to predict all-cause graft failure by predictors used. Predictors: donor characteristics, recipient characteristics and transplant process.

Summary of discrimination by predictor: death-censored graft failure

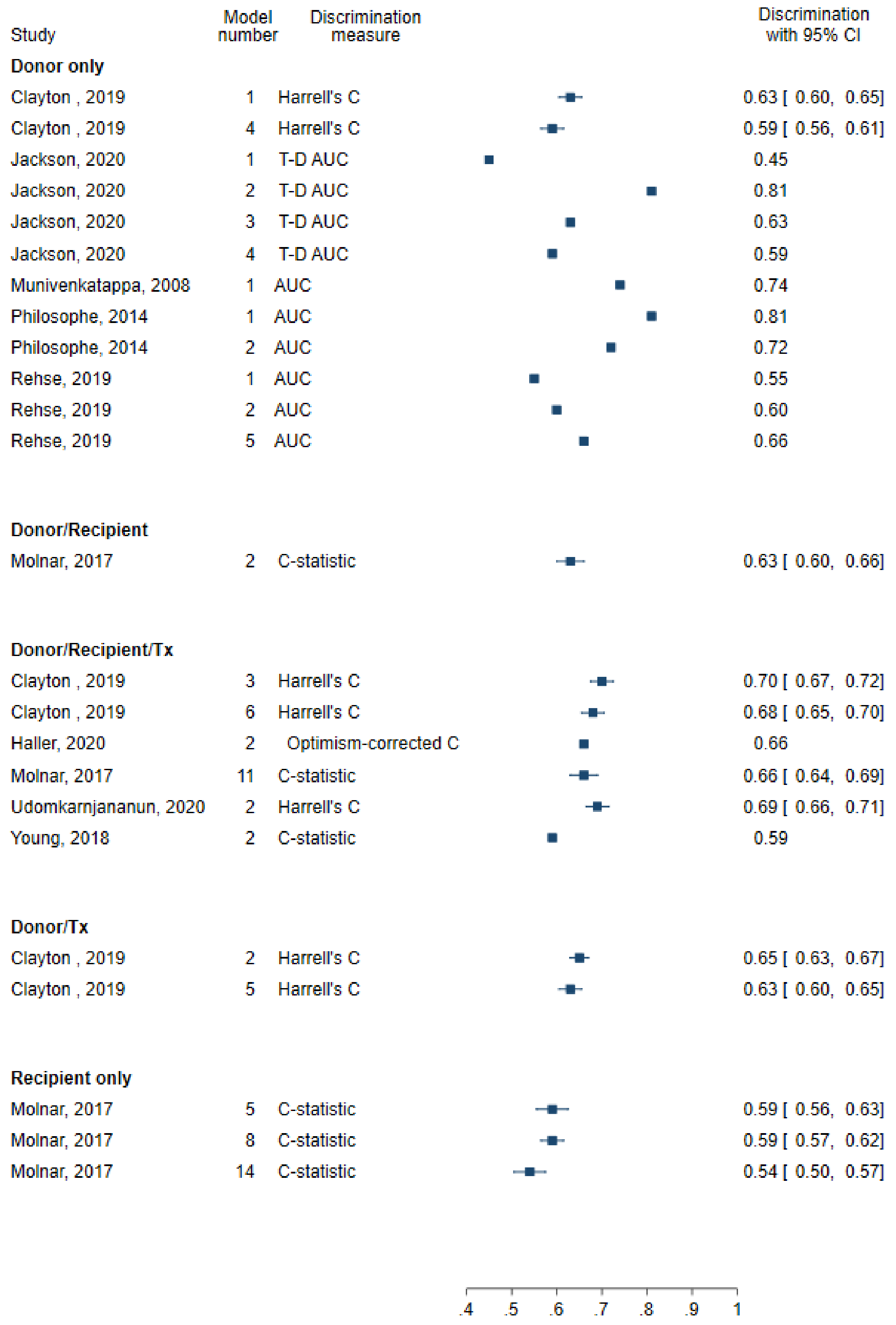

Figure S2: Discrimination of models to predict death-censored graft failure by the types of predictors used. Predictors: donor characteristics, recipient characteristics and transplant process.

Summary of discrimination by predictor: patient survival

Study	Model number	Discrimination measure	Discrimination with 95% CI
**Donor only**			
Calvillo-Arbizu, 2018	2	Harrell's C	0.63
Coca, 2020	1	Harrell's C	0.57
Jassal, 2005	1	C-statistic	0.70
**Donor/Recipient**			
Bae, 2019	1	C-statistic	0.64
Clayton , 2014	2	Harrell's C	0.68 [0.66, 0.70]
Clayton , 2014	3	Harrell's C	0.69 [0.67, 0.71]
Coca, 2020	2	Harrell's C	0.65
Molnar, 2017	1	C-statistic	0.70 [0.67, 0.73]
**Donor/Recipient/Tx**			
Bui, 2019	1	C-statistic	0.70
Bui, 2019	2	C-statistic	0.71
Bui, 2019	3	C-statistic	0.70
Bui, 2019	4	C-statistic	0.71
Haller, 2020	1	Optimism-corrected C	0.77
Molnar, 2017	10	C-statistic	0.68 [0.65, 0.70]
Udomkarnjananun, 2020	1	Harrell's C	0.64 [0.59, 0.68]
**Recipient only**			
Baskin-Bey, 2007	1	C-statistic	0.69
Clayton , 2014	1	Harrell's C	0.67 [0.65, 0.69]
Coca, 2020	3	Harrell's C	0.71
Coca, 2020	4	Harrell's C	0.74
Molnar, 2017	4	C-statistic	0.70 [0.67, 0.72]
Molnar, 2017	7	C-statistic	0.66 [0.63, 0.69]
Molnar, 2017	13	C-statistic	0.70 [0.67, 0.72]

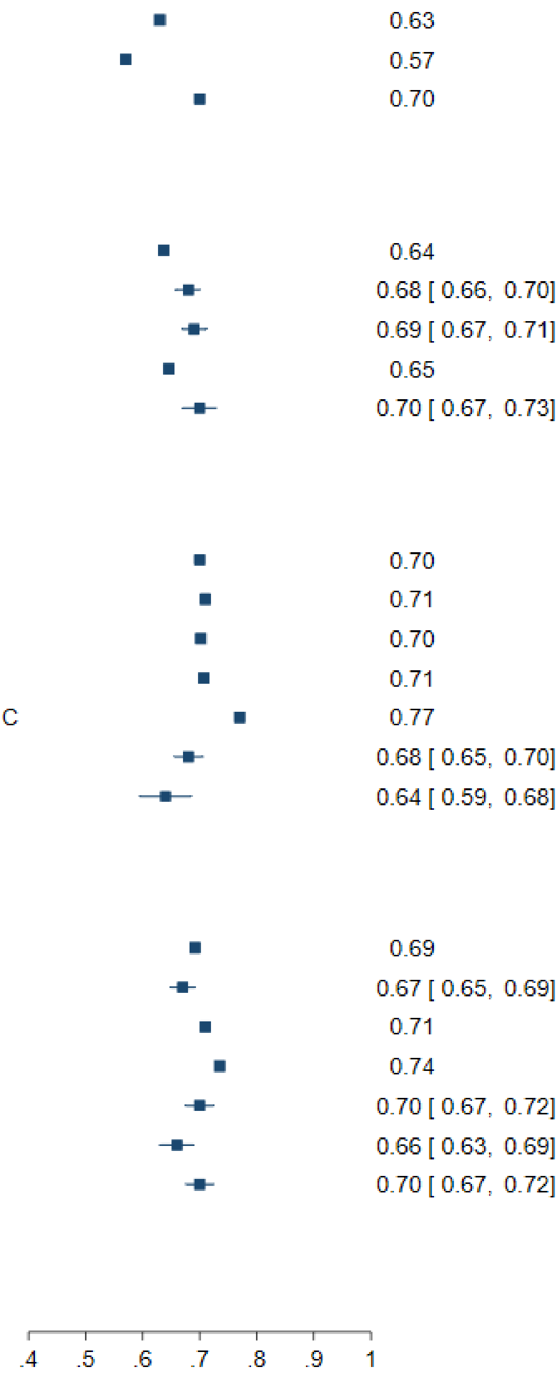

Figure S3: Discrimination of models to predict patient survival by the types of predictors used. Predictors: donor characteristics, recipient characteristics and transplant process.

Summary data: all-cause graft failure

Study	Model number	Model type	Location	Sample size	Predictor type	Discrimination	Value	Calibration	Value(s)/comment
**Kasiske, 2010**	1	Development and validation	US	41363	Donor/ Recipient/ Tx	C-statistic	0.649	Calibration slope	1.04
**Kasiske, 2010**	2	Development and validation	US	41363	Donor/ Recipient/ Tx	C-statistic	~0.636	Calibration slope	1.04
**Massie, 2016**	1	Development and validation	US	106019	Donor/ Recipient	C-statistic	0.59, 95% CI (0.55, 0.62)	None	None
**Molnar, 2017**	3	Development and validation	US	10086	Donor/ Recipient	C-statistic	0.63, 95% CI (0.61, 0.66)	Calibration plot	Adequate
**Molnar, 2017**	6	Development and validation	US	10086	Recipient only	C-statistic	0.61, 95% CI (0.59, 0.63)	Calibration plot	Good
**Rose, 2018**	1	Development and validation	Canada	785	Donor/ Recipient	C-statistic	0.63, 95% CI (0.58, 0.67)	None	None
**Rose, 2018**	2	Development and validation	Canada	785	Donor/ Recipient	C-statistic	0.64, 95% CI (0.61, 0.71)	None	None
**Watson, 2011**	1	Development and validation	United Kingdom	7620	Donor/ Recipient/ Tx	C-statistic	0.62, (SE 0.011)	None	None
**Yang, 2019**	1	Development and validation	US	39108	Donor only	Time-dependent AUC	0.673	None	None

**Yang, 2019**	2	Development and validation	US	39108	Recipient only	Time-dependent AUC	0.742	None	None
**Zhong, 2019**	1	Development and validation	US	156069	Donor/ Recipient	C-statistic	0.652	None	None
**Clayton , 2019**	7	Validation only	Australia and New Zealand	6405	Donor only	Harrell's C	0.60, 95% CI (0.58, 0.62)	None	None
**Clayton , 2019**	10	Validation only	Australia and New Zealand	6405	Donor only	Harrell's C	0.58, 95% CI (0.56, 0.60)	None	None
**Massie, 2016**	2	Validation only	US	69994	Donor only	C-statistic	0.58, 95% CI (0.54, 0.61)	None	None
**Molnar, 2017**	9	Validation only	US	5042	Recipient only	C-statistic	0.57, 95% CI (0.54, 0.59)	None	None
**Molnar, 2017**	12	Validation only	US	5042	Donor/ Recipient/ Tx	C-statistic	0.62, 95% CI (0.60, 0.64)	None	None
**Molnar, 2017**	15	Validation only	US	5042	Recipient only	C-statistic	0.61, 95% CI (0.58, 0.63)	None	None
**Rehse, 2019**	3	Validation only	Germany	416	Donor only	AUC	0.53	None	None
**Rehse, 2019**	4	Validation only	Germany	416	Donor only	AUC	0.54	None	None
**Rehse, 2019**	6	Validation only	Germany	889	Donor only	AUC	0.65	None	None
**Watson, 2011**	2	Validation only	United Kingdom	3050	Donor only	C-statistic	0.63	None	None

**Clayton , 2019**	8	Validation only (adjusted)	Australia and New Zealand	6405	Donor/Tx	Harrell's C	0.62, 95% CI (0.60, 0.64)	None	None
**Clayton , 2019**	9	Validation only (adjusted)	Australia and New Zealand	6405	Donor/ Recipient/ Tx	Harrell's C	0.66, 95% CI (0.64, 0.67)	None	None
**Clayton , 2019**	11	Validation only (adjusted)	Australia and New Zealand	6405	Donor/Tx	Harrell's C	0.61, 95% CI (0.59, 0.63)	None	None
**Clayton , 2019**	12	Validation only (adjusted)	Australia and New Zealand	6405	Donor/ Recipient/ Tx	Harrell's C	0.65, 95% CI (0.63, 0.67)	None	None
**Young, 2018**	1	Validation only (adjusted)	Canada	1299	Donor/ Recipient/ Tx	C-statistic	0.59	None	None

Table S3: Characteristics of models from included studies with all-cause graft failure as an outcome.

Summary data: death-censored graft failure

Study	Model number	Model type	Location	Sample size	Predictor type	Discrimination	Value(s)	Calibration	Value(s)/ comment
**Haller, 2020**	2	Development and validation	Norway	837	Donor/ Recipient/ Tx	Optimism-corrected C-statistic	0.66	Calibration slope	Very good
**Molnar, 2017**	2	Development and validation	US	10086	Donor/ Recipient	C-statistic	0.63, 95% CI (0.60, 0.66)	Calibration slope	Good
**Molnar, 2017**	5	Development and validation	US	10086	Recipient only	C-statistic	0.59, 95% CI (0.56, 0.63)	Calibration slope	Good
**Munivenkatappa, 2008**	1	Development and validation	US	259	Donor only	AUC	0.74	None	None
**Udomkarnjananun, 2020**	2	Development and validation	Thailand	6662	Donor/ Recipient/ Tx	Harrell's C	0.69, 95% CI (0.66, 0.71)	Hosmer-Lemeshow test	5yr: p=0.466 10 yr: p=0.182
**Clayton , 2019**	1	Validation only	Australia and New Zealand	6405	Donor only	Harrell's C	0.63, 95% CI (0.60, 0.65)	None	None
**Clayton , 2019**	4	Validation only	Australia and New Zealand	6405	Donor only	Harrell's C	0.59, 95% CI (0.56, 0.61)	None	None

**Jackson, 2020**	1	Validation only	US	140	Donor only	AUC	0.45	None	None
**Jackson, 2020**	2	Validation only	US	140	Donor only	AUC	0.81	None	None
**Jackson, 2020**	3	Validation only	US	140	Donor only	AUC	0.63	None	None
**Jackson, 2020**	4	Validation only	US	140	Donor only	AUC	0.59	None	None
**Molnar, 2017**	8	Validation only	US	5042	Recipient only	C-statistic	0.59, 95% CI (0.57, 0.62)	None	None
**Molnar, 2017**	11	Validation only	US	5042	Donor/ Recipient/ Tx	C-statistic	0.66, 95% CI (0.64, 0.69)	None	None
**Molnar, 2017**	14	Validation only	US	5042	Recipient only	C-statistic	0.54, 95% CI (0.50, 0.57)	None	None
**Philosophe, 2014**	1	Validation only	US	140	Donor only	AUC	0.81	None	None
**Philosophe, 2014**	2	Validation only	US	56	Donor only	AUC	0.72	None	None
**Rehse, 2019**	1	Validation only	Germany	416	Donor only	AUC	0.55	None	None
**Rehse, 2019**	2	Validation only	Germany	416	Donor only	AUC	0.6	None	None
**Rehse, 2019**	5	Validation only	Germany	889	Donor only	AUC	0.66	None	None

**Clayton , 2019**	2	Validation only (adjusted)	Australia and New Zealand	6405	Donor/Tx	Harrell's C	0.65, 95% CI (0.63, 0.67)	None	None
**Clayton , 2019**	3	Validation only (adjusted)	Australia and New Zealand	6405	Donor/ Recipient/ Tx	Harrell's C	0.70, 95% CI (0.67, 0.72)	None	None
**Clayton , 2019**	5	Validation only (adjusted)	Australia and New Zealand	6405	Donor/Tx	Harrell's C	0.63, 95% CI (0.60, 0.65)	None	None
**Clayton , 2019**	6	Validation only (adjusted)	Australia and New Zealand	6405	Donor/ Recipient/ Tx	Harrell's C	0.68, 95% CI (0.65, 0.70)	None	None
**Young, 2018**	2	Validation only (adjusted)	Canada	1299	Donor/ Recipient/ Tx	C-statistic	0.59	None	None

Table S4: Characteristics of models from included studies with the outcome death-censored graft failure.

Summary data: patient survival

Study	Model number	Model type	Location	Sample size	Predictor type	Discrimination	Value(s)	Calibration	Value(s)/ comment
**Bae, 2019**	1	Development and validation	US	120818	Donor/ Recipient	C-statistic	0.637	None	None
**Baskin-Bey, 2007**	1	Development and validation	US	47535	Recipient only	C-statistic	0.692	None	None
**Bui, 2019**	1	Development and validation	US	72839	Donor/ Recipient/ Tx	C-statistic	0.7	None	None
**Bui, 2019**	2	Development and validation	US	72839	Donor/ Recipient/ Tx	C-statistic	0.71	None	None
**Bui, 2019**	3	Development and validation	US	53242	Donor/ Recipient/ Tx	C-statistic	0.702	None	None
**Bui, 2019**	4	Development and validation	US	53242	Donor/ Recipient/ Tx	C-statistic	0.707	None	None
**Haller, 2020**	1	Development and validation	Norway	837	Donor/ Recipient/ Tx	Optimism-corrected C-statistic	0.77	Calibration plot	Good
**Jassal, 2005**	1	Development and validation	Canada	6324	Donor only	C-statistic	0.7	None	None
**Molnar, 2017**	1	Development and validation	US	10086	Donor/ Recipient	C-statistic	0.70, 95% CI (0.67, 0.73)	Calibration plot	Good

**Molnar, 2017**	4	Development and validation	US	10086	Recipient only	C-statistic	0.70, 95% CI (0.67, 0.72)	Calibration plot	Adequate
**Udomkarnjananun, 2020**	1	Development and validation	Thailand	6662	Donor/ Recipient/ Tx	Harrell's C	0.64, 95% CI (0.59, 0.68)	Hosmer-Lemeshow test	5 yr: p=0.252 10 yr: p=0.851
**Calvillo-Arbizu, 2018**	2	Validation only	Spain	2734	Donor only	Harrell's C	0.63	None	None
**Clayton , 2014**	1	Validation only	Australia and New Zealand	4983	Recipient only	Harrell's C	0.67, SE 0.011	None	None
**Coca, 2020**	1	Validation only	Spain	935	Donor only	Harrell's C	0.57	None	None
**Coca, 2020**	3	Validation only	Spain	935	Recipient only	Harrell's C	0.71	None	None
**Molnar, 2017**	7	Validation only	US	5042	Recipient only	C-statistic	0.66, 95% CI (0.63, 0.69)	None	None
**Molnar, 2017**	10	Validation only	US	5042	Donor/ Recipient/ Tx	C-statistic	0.68, 95% CI (0.65, 0.70)	None	None
**Molnar, 2017**	13	Validation only	US	5042	Recipient only	C-statistic	0.70, 95% CI (0.67, 0.72)	None	None
**Clayton , 2014**	2	Validation only (adjusted)	Australia and New Zealand	4983	Donor/ Recipient	Harrell's C	0.68, SE 0.011	None	None

**Clayton , 2014**	3	Validation only (adjusted)	Australia and New Zealand	4983	Donor/ Recipient	Harrell's C	0.69, SE 0.011	None	None
**Coca, 2020**	2	Validation only (adjusted)	Spain	935	Donor/ Recipient	Harrell's C	0.646	None	None
**Coca, 2020**	4	Validation only (adjusted)	Spain	935	Recipient only	Harrell's C	0.735	None	None

Table S5: Characteristics of models from included studies with patient survival as the outcome.

Summary data: other

Study	Model number	Model type	Location	Sample size	Predictor type	Discrimination	Value(s)	Calibration	Value(s)/comment
**Tiong, 2009**	1	Development and validation	US	20085	Donor/ Recipient/Tx	C-statistic	0.71	Calibration plot	Good
**Calvillo-Arbizu, 2018**	1	Validation only	Spain	2734	Donor only	Harrell's C	0.56	None	None

Table S6: Characteristics of models from included studies for graft failure with no information on how death was handled.
